# *Burkholderia pseudomallei* Known Siderophores and Hemin Uptake Are Dispensable for Lethal Murine Melioidosis

**DOI:** 10.1371/journal.pntd.0001715

**Published:** 2012-06-26

**Authors:** Brian H. Kvitko, Andrew Goodyear, Katie L. Propst, Steven W. Dow, Herbert P. Schweizer

**Affiliations:** Department of Microbiology, Immunology and Pathology, Rocky Mountain Regional Center of Excellence for Biodefense and Emerging Infectious Diseases Research, Colorado State University, Fort Collins, Colorado, United States of America; Yale University School of Medicine, United States of America

## Abstract

*Burkholderia pseudomallei* is a mostly saprophytic bacterium, but can infect humans where it causes the difficult-to-manage disease melioidosis. Even with proper diagnosis and prompt therapeutic interventions mortality rates still range from >20% in Northern Australia to over 40% in Thailand. Surprisingly little is yet known about how *B. pseudomallei* infects, invades and survives within its hosts, and virtually nothing is known about the contribution of critical nutrients such as iron to the bacterium's pathogenesis. It was previously assumed that *B. pseudomallei* used iron-acquisition systems commonly found in other bacteria, for example siderophores. However, our previous discovery of a clinical isolate carrying a large chromosomal deletion missing the entire malleobactin gene cluster encoding the bacterium's major high-affinity siderophore while still being fully virulent in a murine melioidosis model suggested that other iron-acquisition systems might make contributions to virulence. Here, we deleted the major siderophore malleobactin (*mba*) and pyochelin (*pch*) gene clusters in strain 1710b and revealed a residual siderophore activity which was unrelated to other known *Burkholderia* siderophores such as cepabactin and cepaciachelin, and not due to increased secretion of chelators such as citrate. Deletion of the two hemin uptake loci, *hmu* and *hem*, showed that Hmu is required for utilization of hemin and hemoglobin and that Hem cannot complement a Hmu deficiency. Prolonged incubation of a *hmu hem* mutant in hemoglobin-containing minimal medium yielded variants able to utilize hemoglobin and hemin suggesting alternate pathways for utilization of these two host iron sources. Lactoferrin utilization was dependent on malleobactin, but not pyochelin synthesis and/or uptake. A *mba pch hmu hem* quadruple mutant could use ferritin as an iron source and upon intranasal infection was lethal in an acute murine melioidosis model. These data suggest that *B. pseudomallei* may employ a novel ferritin-iron acquisition pathway as a means to sustain *in vivo* growth.

## Author Summary

*Burkholderia pseudomallei* is the etiologic agent of melioidosis, a multifaceted deadly and difficult to treat disease of equatorial regions of the world. Disease manifestations range from acute infections to long term chronic infections. The factors by which this bacterium causes disease are not yet well understood. Studies thus far focused on elucidation of the roles of traditional virulence factors such as secreted proteins and exopolysaccharides, but virtually nothing is known about the roles of nutrient acquisition systems in *B. pseudomallei*'s survival in its mammalian hosts. One nutrient that is essential for bacterial metabolism and pathogenicity is iron. As free iron is not readily available in nature, bacteria developed numerous mechanisms for iron acquisition from abiotic and biotic sources. These mechanisms include siderophores and hemin/hemoglobin utilization systems, and it is therefore not too surprising that mutants defective in these systems are often impaired in virulence. In this study we show that defined *B. pseudomallei* mutants defective in siderophore and hemin/hemoglobin utilization systems remain fully lethal in a murine melioidosis model and present evidence for *in vitro* ferritin-iron acquisition which may be one or perhaps the main means by which this pathogen sustains *in vivo* growth.

## Introduction

*Burkholderia pseudomallei* is a Gram-negative bacterial pathogen that normally survives as a saprophyte in soil and water, but is also capable of infecting most mammals and causing serious infections resulting in the multifaceted disease melioidosis [1]–[7]. Even with rapid diagnosis and prompt and aggressive treatment the fatality rate for melioidosis patients still ranges from 10–20% in Australia to over 40% in Thailand. *B. pseudomallei* is considered an emerging pathogen and infections have been increasingly reported in many countries in tropical and subtropical regions of the world [8]–[12].

Iron is essential for bacteria, yet in almost any abiotic or biotic environment bacteria are confronted with levels of soluble iron too low to sustain growth [13]. The two main strategies used by Gram-negative bacteria to acquire biotic iron are uptake of iron-siderophore complexes and uptake of heme [14]. Because of the necessity for iron uptake, siderophore dependent uptake mechanisms are considered virulence factors and corresponding mutants are severely attenuated in animal models of infection [15]–[20].

In *Burkholderia* species, iron acquisition mechanisms have been best characterized in members of the *Burkholderia cepacia* complex (Bcc) [21]. These bacteria produce as many as four different siderophores (ornibactin, pyochelin, cepabactin and cepaciachelin). In addition, Bcc bacteria possess mechanisms for acquiring iron from heme and ferritin [21], [22].

Very little is known about iron acquisition mechanisms in *B. pseudomallei*. The bacterium produces a hydroxamate-type siderophore, malleobactin, that can remove iron from lactoferrin and transferrin, allowing this bacterium to grow under iron-limiting conditions [23]–[25]. Genome-wide microarray expression and whole genome sequence analyses identified genes encoding a number of other iron acquisition systems such as a pyochelin (*pch*) gene cluster, a heme uptake locus (*hmu*) and plasma membrane iron transporters [26]–[29]. Despite the recognized importance of iron acquisition systems, no data have been published about the contribution of any of these to *B. pseudomallei* virulence. There is evidence that iron availability influences colony morphology [30], [31](our unpublished results), a not well understood characteristic of *B. pseudomallei* that affects virulence and antimicrobial susceptibility [30], [31].

We previously discovered that when compared to other sequenced strains the clinical isolate 708a contains a large (>130 kb) genomic deletion [32]. This deleted region includes the *amrAB-oprA* efflux pump operon which explains the gentamicin sensitivity of 708a. The >90 gene region also contains numerous other genes that may be pertinent for *B. pseudomallei*'s physiology and pathogenesis. Of note is absence of the complete malleobactin biosynthetic gene cluster. Despite lack of this gene cluster and, presumably, malleobactin, 708a caused human melioidosis and was fully lethal in the acute murine melioidosis model [32]. This finding was somewhat surprising because in *Pseudomonas aeruginosa* the analogous siderophore pyoverdine is essential for infection and full virulence [15]. Similarly, *B. cenocepacia* mutants lacking ornibactin showed significantly reduced virulence [17].

As we could not rule out the presence of mutations in 708a that compensated *in vivo* for the loss of malleobactin synthesis, we sought to elucidate the contribution of this siderophore and other annotated iron acquisition systems, including pyochelin synthesis and uptake and hemin utilization to virulence in isogenic *B. pseudomallei* mutants. Our studies revealed that none of these systems is required for lethality in an acute murine melioidosis model and provided evidence for a ferritin-iron utilization system.

## Materials and Methods

### Bacterial strains and growth conditions

*B. pseudomallei* strains used in this study are listed in **Table 1**. All procedures involving *B. pseudomallei* were performed in a Select Agent approved Biosafety Level 3 (BSL3) facility using Select Agent compliant procedures and protocols. Unless noted otherwise, bacteria were routinely grown at 37°C in Lennox LB broth [33] or Lennox LB agar plates (MO BIO Laboratories, Carlsbad, CA). For *Escherichia coli*, antibiotics and other media additives were used at the following final concentrations: ampicillin (Amp), 100 µg/ml; kanamycin (Km), 40 µg/ml; zeocin (Zeo), 25 µg/ml; gentamicin (Gm), 10 µg/ml; 5-bromo-4-chloro-3-indolyl-β-D-galactopyranoside (X-gal), 40 µg/ml; 5-bromo-4-chloro-3-indolyl glucuronide (X-gluc), 40 µg/ml; and diaminopimelic acid (DAP), 200–400 µg/ml. For AmrAB-OprA pump expressing *B. pseudomallei* strains antibiotic concentrations used were 500–1000 µg/ml Km and 1000–2000 µg/ml Zeo. For *B. pseudomallei* Δ(*amrRAB-oprA*) strains, these were adjusted to 35–50 µg/ml Km; 25–50 µg/ml Zeo; and 10–30 µg/ml Gm. Antibiotics were purchased from EMD Biosciences, San Diego, CA (Gm); Sigma, St. Louis, MO (Amp and Km); and Invitrogen, Carlsbad, CA (Zeo). DAP (LL−, DD−, and meso-isomers) was obtained from Sigma. X-gal and X-gluc were purchased from Gold Biotechnology, St. Louis, MO. Arabinose or rhamnose were used to induce *B. pseudomallei* expression plasmids at final concentrations of 0.2–0.5%.

**Table 1:** *B. pseudomallei* strains used in this study.

Strain	Relevant features^a^	Mutation acronym	Reference/source
708a	Clinical isolate; carries a deletion of the malleobactin synthesis gene cluster and the *amrRAB-oprA* efflux operon		[32]
1710b	Clinical isolate		Sharon Peacock
Bp74	1710b Δ(*amrRAB-oprA*)::*FRT*-*ble-FRT*; Zeo^R^		[35]
Bp327	1710b Δ(*mbaS-mbaF*)::*FRT* b	ΔMBA	This study
Bp338	1710b Δ(BURPS1710b_2054-BURPS1710b_2155)::*FRT* c	Δ141-kb	This study
Bp416	1710b Δ(BURPS1710b_2054-BURPS1710b_2155)::*FRT* Δ*fptA*	Δ141-kb Δ*fptA*	This study
Bp447	1710b Δ*pchA*::*FRT*	Δ*pchA*	This study
Bp448	1710b Δ(*mbaS-mbaF*)::*FRT* Δ*pchA*::*FRT*	ΔMBA Δ*pchA*	This study
Bp449	1710b Δ(BURPS1710b_2054-BURPS1710b_2155)::*FRT* Δ*pchA*	Δ141-kb Δ*pchA*	This study
Bp486	1710b Δ(*pchBA*::*FRT*-*nptII*-*FRT*); Km^R^	Δ*pchA-B*	This study
Bp487	1710b Δ(BURPS1710b_2054-BURPS1710b_2155)::*FRT* Δ(*pchBA*::*FRT*-*nptII*-*FRT*); Km^R^	Δ141-kb Δ*pchA-B*	This study
Bp515	1710b Δ(*pchA*-*fptA*)::*FRT*	ΔPCH	This study
Bp516	1710b Δ(BURPS1710b_2054-BURPS1710b_2155)::*FRT* Δ(*pchA*-*fptA*)::*FRT*	Δ141-kb ΔPCH	This study
Bp549	1710b Δ(BURPS1710b_2054-BURPS1710b_2155)::*FRT* Δ(*pchA*-*fptA*)::*FRT* Δ(*hmuV-*BURPS1710b_A1781)::*FRT*	Δ141-kb ΔPCH ΔHMU	This study
Bp568	1710b Δ(BURPS1710b_2054-BURPS1710b_2155)::*FRT* Δ(*pchA*-*fptA*)::*FRT* Δ(*hmuV-*BURPS1710b_A1781)::*FRT*, *Δ*(*btuC-*BURPS1710b_3209)::*FRT*	Δ141-kb ΔPCH ΔHMU ΔHEM	This study
Bp576	Hemoglobin-adapted Bp568	Δ141-kb ΔPCH ΔHMU ΔHEM	This study

a Abbreviations: *FRT*, Flp recombinase target; Km, kanamycin; R, resistance; S, sensitive/susceptible; of Zeo, zeocin.

b *mbaS* is annotated as *psbS* in the 1710b genome annotation (GenBank accession number NC_007434.1).

c All strains containing Δ(BURPS1710b_2054–BURPS1710b_2155)::*FRT* are aminoglycoside susceptible because the deletion of the genes encoding the AmrRAB-OprA efflux pump.

### Recombinant DNA techniques

*E. coli* DH5α was used for plasmid maintenance and construction. Plasmid DNA was prepared using the GeneJET plasmid miniprep kit from Fermentas Life Sciences (Glen Burnie, MD). Genomic DNA was prepared using the Puregene genomic DNA purification kit from Gentra Systems (Qiagen, Valencia, CA). DNA purification of enzyme reactions and DNA gel extractions were conducted using the GenElute gel extraction kit from Sigma Life Science. PCR DNA polymerases, restriction enzymes and DNA modification enzymes were purchased from New England Biolabs (Ipswich, MA) and used essentially according to the manufacturer's recommendations. PCR was typically conducted with either *Taq* polymerase or platinum HiFi *Taq* polymerase. DNA blunting reactions were conducted with T4 polymerase, DNA dephosphorylation reactions were performed with calf intestinal alkaline phosphatase and DNA ligations were conducted with T4 DNA ligase. Southern analysis was performed using the NEBlot Phototope and Phototope-Star chemiluminescent labeling and detection kits from New England Biolabs following the manufacturer recommendations and using standard capillary transfer and blotting procedures [34]. The QuikChange site-directed mutagenesis kit from Stratagene (Santa Clara, CA) was used according to the manufacturer's recommendations.

Competent *E. coli* cells were prepared and transformed by the rubidium chloride method essentially as described by [34]. T4 DNA ligation reactions were typically drop-dialysed on 25 mm diameter filters (MF type, VS filter, mean pore size 0.025 µm from Millipore (Billerica, MA) for 20 min prior to use in transformations.

Plasmids were introduced into *B. pseudomallei* by electroporation which was conducted essentially as previously described [35]. Briefly,overnight cultures were washed several times in 300 mM sucrose and concentrated 10-fold. Aliquots (100 µl) were electroporated using a 2 mm gap disposable electroporation cuvette at 2.5 kV with a GenePulser Xcell from Bio-Rad (Hercules, CA). Cells were recovered after electroporation and outgrown for 1 h in LB prior to plating on appropriate selective media.

*E. coli* RHO3 was used for conjugation of plasmids into *B. pseudomallei* [36]. Conjugations were performed essentially as previously described [36]. Briefly, overnight cultures were washed and concentrated 5-fold. Equal parts of each parent strain were mixed and applied to sterile cellulose acetate filters along with parental controls on LB plates augmented with 200–400 µg/ml DAP. After overnight incubation, cells were recovered from the membrane by centrifugation, washed, and plated on appropriate selective media lacking DAP.

### Oligonucleotides

Oligonucleotides were purchased from Integrated DNA Technologies, Coralville, IA, and are listed in **Table S1**.

### Plasmids

A comprehensive list of plasmids used in this study is provided in **Table S2**. Individual plasmids were constructed as follows. To create pEXGm5B, the 0.9-kb fragment of pPS856 [37] was released by *Xba*I digest of unmethylated DNA prepared from *E. coli* JM110 [38] and ligated with the *Spe*I+*Xba*I digested backbone of pEXKm5 [36]. pFKm4 was constructed by removing undesired *Xba*I and *Spe*I sites from pFKM2 and adding a *Pac*I site by using QuikChange site-directed mutagenesis (Stratagene, La Jolla, CA).

### Strain construction

*B. pseudomallei* deletions strains were constructed using previously described methods [36]. Briefly, 500–1500 bp of genomic DNA flanking the desired deletion region were PCR amplified and cloned separately into a TA cloning vector, usually pCR2.1 (Invitrogen) or pGEM-T Easy (Promega, Madison, WI). The cloned DNA fragments were released by digestion with appropriate restriction enzymes and joined by T4 ligation prior to *Eco*RI digestion and cloning into pEXGm5B, a Gm^R^ derivative of the dual counter-selection allelic exchange vector pEXKm5 [36]. A *FRT*-*nptII*-*FRT* Km^R^ cassette was then ligated between the two flanking DNA segments. Plasmid pEXGm5B deletion constructs were introduced into *B. pseudomallei* by conjugation using the *E. coli* RHO3 mobilizer strain and metabolic counter-selection [36]. Km^R^ merodiploids were selected and subsequently resolved using sucrose counter-selection, I-*Sce*I counter-selection, or both strategies to recover the desired deletion mutants [36]. For difficult to isolate mutations YT-sucrose plates were incubated for three to ten days at room temperature and then re-struck to isolation from white bordered colonies onto fresh YT-sucrose plates. The Km^R^ marker was removed using Flp recombinase [35]. Mutations were confirmed by genomic Southern analysis or PCR followed by sequencing. The construction of individual strains is detailed in Text S1.

### Low-iron medium and siderophore testing

Low-iron media were obtained by deferration with Chelex 100 resin and was prepared and stored in plastic-ware to prevent reintroduction of iron. Trypticase soy broth filtrate, chelex-treated (TSBFC) media is a modification of TSBDC [28], [39]. TSBFC consisted (per L) of 30 g of trypticase soy broth, 7.35 g glutamic acid, 12.5 ml of 80% (w/v) glycerol, with the pH adjusted to 6.0 with NaOH. We found that siderophore halo formation on CAS plates was more reproducible by growth on pH 6.0 media. The medium was autoclaved and allowed to cool to room temperature before addition of 30 g autoclaved Chelex 100 resin and agitation for 24 h at room temperature. Chelex treatment raised the pH slightly. Chelex was filtered from the media with a plastic funnel and Whatman 541 filter paper. The filtrate was then sterilized with a 0.2 micron vacuum filtration unit.

### CAS assays

Liquid Chrome Azurol S (CAS) siderophore assay solution was prepared as previously described [40]. 5-sufosalicyclic acid iron shuttle solution was prepared separately and added prior to use. For quantitative measurement of siderophores in culture supernatant, low iron cultures were incubated typically overnight at 37°C with aeration, pelleted and 100 µl supernatant was added to 900 µl CAS assay solution. Mixtures were allowed to incubate for 30 min at room temperature prior to measuring change in A_630 nm_ compared to an uninoculated media control. Measurements were adjusted for cell density by measuring the OD_600 nm_ of a 1∶10 diluted TSBFC culture. CAS plates were prepared as described [41]. For 600 ml of CAS agar the following solutions were prepared. Solution 1 consisted of Parts A and B. For preparation of Part A, 35 mg CAS were dissolved in 30 ml of deionized water which was then mixed with 6.2 ml of 1 mM FeCl_3_·H_2_O in 10 mM HCl. Part B consisted of 47 mg hexadecyltrimethy-ammonium bromide (HDTMA) in 24 ml of deionized water. Parts A and B were then combined to make solution 1 which was then autoclaved. For Solution 2, 1.62 g sodium succinate, 1.68 g casamino acids, 0.43 g Na_2_SO_4_ and 5.44 g PIPES were dissolved in 540 ml of deionized water and the pH adjusted to 6.0 with NaOH. After addition of 9 g of agar Solution 2 was autoclaved. After cooling Solution 2 to 50°C, Solution 1 was added slowly and with mixing. For preparation of CAS plates 30 ml of this mixture was dispensed into a 100 mm Petri dish. These plates were much less likely to oxidize during preparation than those prepared using the original recipe described by Schwyn and Neilands, [42].

### Pyochelin extraction and mass spectrometry

TSBFC overnight cultures (1–3 ml) were harvested in a microcentrifuge by centrifugation at full speed for 1 min at room temperature and supernatants were sterilized with 0.2 micron syringe filter units. The supernatants were acidified to pH 1–2 with concentrated HCl and then extracted three times with five supernatant volumes of ethyl acetate. Ethyl acetate extractions were combined and dried under N_2_ prior to re-suspension in 1∶10 supernatant volumes of methanol. Mass spectrometry was conducted at the Colorado State University Biomolecular Analysis Core with an Agilent 1200 series liquid chromatograph interfaced with the Agilent 6520 quadrupole/time-of-flight for these analyses. The mass spectrometer interface was the Chip-cube nanoflow interface using a G4240-65001 chip chromatography column (40 nL enrichment column with 80 Å, 75 µm×43 mm C18 packing) operated at 0.6 µl/min flow rate. The chromatography gradient used water with 0.1% formic acid and 90% acetonitrile. The nanoelectrospray source was operated in positive ion mode at 2000 V with 5 liters/min drying gas N_2_ at 325°C.

### Determination of citrate levels in cell culture supernatants

To determine citrate concentrations in cell-free culture supernatants, TSBFC cultures were pelleted by centrifugation as described above and supernatants were sterilized with a 0.2 micron syringe filter unit. The supernatant was deproteinated by processing with a Millipore Ultrafree −15 centrifugal filter device, biomax-5K with a 5 kDa molecular weight cutoff. Uninoculated media and culture supernatant was tested with the Citrate Assay Kit from BioVision Incorporated (Mountain View, CA) according to the manufacturer's recommendations.

### Iron source utilization experiments

For testing iron source utilization, 500 ml of M9 medium [34] was Chelex treated by agitation for 5 h with 5 g/L of Chelex 100. The mixture was filtered and then sterilized with a 0.2 micron vacuum filter unit. M9-Chelex treated media were inoculated with bacteria and incubated overnight at 37°C with aeration. They were then sub-cultured 1∶10 into M9 media containing 10 µM porcine hemin (prepared as filter-sterilized 10 mM stock in 1 M NaOH), 2.5 µM porcine hemoglobin (prepared as filter-sterilized 1 mM stock in dH_2_O), 1 µM human recombinant holo-lactoferrin (prepared as filter-sterilized 10 µM stock in phosphate buffered saline, pH 7.4) or 10 µg/ml equine ferritin (prepared as 10 mg/ml stock in sterile saline immediately prior to use) and 200 µM 2,2′-dipyridyl (100 µM for noted experiments)(prepared as 50 mM stock solution in ethanol) to chelate residual inorganic iron. For monitoring bacterial growth using a Synergy HT Multi-Mode Microplate Reader (BioTek, Winooski, VT), 200 µl samples of the subcultures were dispensed into wells of a 96-well flat-bottom plate (Corning Inc. 3603 plates, Corning, NY). Plates were incubated at 37°C with constant shaking at 200 rpm and the optical density at 600 nm was read every hour for up to 96 h.

### Animal infection experiments

Ethics Statement: Animal experiments were performed in strict accordance with the recommendations in the Guide for the Care and Use of Laboratory Animals of the National Institutes of Health. The protocol was approved by the Colorado State University Institutional Animal Care and Use Committee (Permit Number: 10-1736A). Specific-pathogen-free BALB/c mice were purchased from Jackson Laboratories (Bar Harbor, ME). All mice used in experiments were female and 4–6 weeks of age at the time of infection. Animals were housed in microisolator cages under pathogen free conditions. All experiments involving animals were approved by the Institutional Animal Care and Use Committee at Colorado State University. Animal infections were performed as described previously [43]. Briefly, prior to each challenge study, glycerol stocks stored at −80°C were thawed and bacteria diluted in PBS. For intranasal (i.n.) inoculation, mice were anesthetized by intraperitoneal (i.p.) injection with ketamine (100 mg/kg) (Vedco, Saint Joseph, MO) and xylazine (10 mg/kg) (Ben Venue Labs, Bedford, OH). Mice were infected i.n. with a total volume of 20 µ of bacterial inoculum (10 µl per nostril). The LD_50_ for *B. pseudomallei* strain 1710b was found to be similar to the LD_50_ previously reported for *B. pseudomallei* strain 1026b (approximately 900 CFU) [43]. All mice were challenged with an experimentally determined LD_100_ dose of approximately 3×10^3^ CFU (∼4 LD_50_). Inoculum titers for each experiment were confirmed by plating the inoculum on LB or Trypticase soy agar medium (TSA; BD Bioscience, Sparks, MD). Euthanasia endpoints used in this study included hunched posture with decreased movement or response to stimuli, development of respiratory distress, or loss of >15% body weight.

### Determination of bacterial organ burden

Bacterial burden was determined in lung liver and spleen tissues as described previously [43]. Briefly, upon reaching an euthanasia endpoint mice were euthanized and lung, liver and spleen tissues were collected separately. Tissues were placed in 4 ml sterile PBS, and organs were homogenized using a Stomacher 80 Biomaster (Seward, Bohemia, NY). Homogenates were serially diluted in PBS and plated on TSA plates. Plates were incubated at 37°C for 48 hours, colonies were counted and titers expressed as CFU/organ. The limit of detection in organ homogenates was 40 CFU/organ.

### Statistical analysis

Statistical analyses were performed using Prism 5.0 software (Graph Pad, La Jolla, CA). Survival times were analyzed by Kaplan-Meier analysis, followed by the log-rank test. Differences in organ bacterial burdens were compared using a two-tailed Student's T-test. Differences for survival experiments were considered statistically significant for *p*-values≤0.013 after performing the Bonferroni correction for multiple comparisons. For comparison of bacterial burden, differences were considered statistically significant for *p*<0.05.

## Results

### Malleobactin synthesis mutants exhibit residual siderophore activity

**Figure 1:**
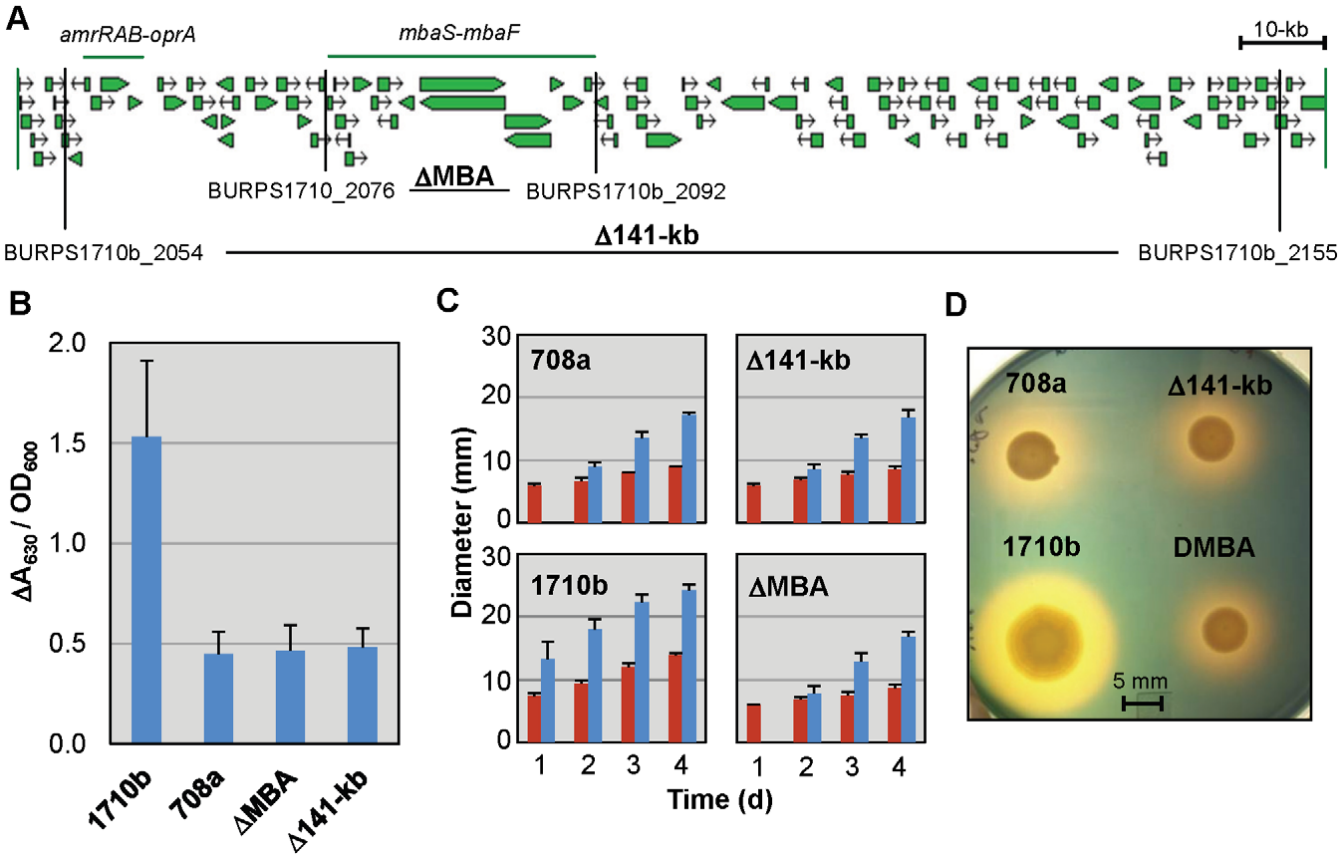
*B. pseudomallei* 1710b malleobactin mutant strains mimic the siderophore phenotypes of the *B. pseudomallei* 708a clinical isolate. **A**. *Burkholderia* GBrowse map of the *B. pseudomallei* 1710b genomic region corresponding to the extent of the deletion found in the *B. pseudomallei* 708a clinical isolate [32], [62]. The extents of the *amrRAB-oprA* genes ecoding the AmrAB-OprA efflux pump and AmrR repressor and the malleobactin synthesis gene cluster (*mbaS-mbaF*) are noted with horizontal green lines. Vertical black lines and gene locus numbers indicate the borders of 1710b genomic region deletions constructed in this study. The shorthand nomenclatures for strains indicating the genomic region deletions contained in them are bolded. **B.** Quantitative CAS siderophore assays indicate similar amounts of secondary siderophore production by 1710b malleobactin minus strains and *B. pseudomallei* 708a. Supernatants from overnight cultures grown in low-iron TSBFC medium were tested by quantitative CAS assays for siderophore production adjusted for cell density by OD_600_ of a 1∶10 dilution. Means and standard deviations of two measurements each from three independent experiments are shown. **C.** Bacterial colony and CAS halo diameters were measured daily for 4 days on CAS agar plates spotted and incubated as described above. Red bars indicate colony diameter and blue bars halo diameter. Means and standard deviations of two measurements each from three independent experiments are shown. **D.** CAS plate assays indicate similar secondary siderophore production by 1710b malleobactin deficient strains and *B. pseudomallei* 708a. Five µl samples of overnight cultures grown in low-iron TSBFC medium were spotted onto CAS agar plates and incubated at 37°C for 4 days prior to photographing.

To exclude the presence of compensatory mutations that could perhaps allow strain 708a to overcome iron-acquisition constraints posed by deletion of the malleobactin synthesis gene cluster, we recreated the 141-kb genomic deletion in the defined strain 1710b genetic background. This strain was mainly chosen because we used it as the comparator for defining the extent of the 141-kb deletion in strain 708a as its genome sequence is known and annotated, and strong siderophore production and ease of genetic manipulation. In addition, we isolated a specific deletion of the malleobactin synthesis region to allow us to distinguish the contribution of malleobactin from that of other genes in the 141-kb region to siderophore production and/or virulence. Gene replacement was used to introduce the 141-kb Δ(*BURPS1710b_2054–BURPS1710b_2155*) (**Figure 1A**) into strain Bp74, a Δ(*amrRAB-oprA*)::*FRT*-*ble*-*FRT* (zeocin resistant) derivative of 1710b [35] to allow for more effective antibiotic selection. The Δ(*amrRAB-oprA*)::*FRT*-*ble*-*FRT* region is deleted from the resulting Δ(*BURPS1710b_2054–BURPS1710b_2155*)(hereafter Δ141-kb) strain (Bp338). The 31-kb Δ(*mbaS-mbaF*) (**Figure 1A**) was introduced into 1710b to yield ΔMBA (Bp327). To assess siderophore production, strains 708a, 1710b, Δ141-kb and ΔMBA were grown in chelex-treated tryptic soy broth (TSBFC), a low iron growth medium. Siderophore levels in cell free culture supernatants were assessed using a quantitative chrome azurol S (CAS) total siderophore assay and CAS agar plates (**Figure 1B and D**). While total siderophore production was considerably reduced when compared to 1710b, strains 708a, ΔMBA and Δ141-kb behaved similarly in terms of timing of secondary (non-malleobactin) halo formation and appearance. After 1 day, 1710b showed a well-defined halo, but strains 708a, ΔMBA and Δ141-kb produced a barely noticeable halo. After 4 days, all strains produced halos, but the halos of malleobactin plus and minus strains exhibited distinct appearances (**Figure 1D**). The halo formed by 1710b is yellow in color with a distinct border while the halos of strains defective in malleobactin synthesis appear more red with diffuse borders Variations in halo appearance have been noted previously in response to production of different siderophores [25]. We also found that siderophore halo formation on CAS plates varied over a narrow pH range from pH 6.8 to 6.0. The largest malleobactin-dependent halos were formed at pH 6.8 but the largest malleobactin-independent halos were formed at pH 6.0.

### Pyochelin production is dependent on an intact *pch-ftpA* locus

**Figure 2:**
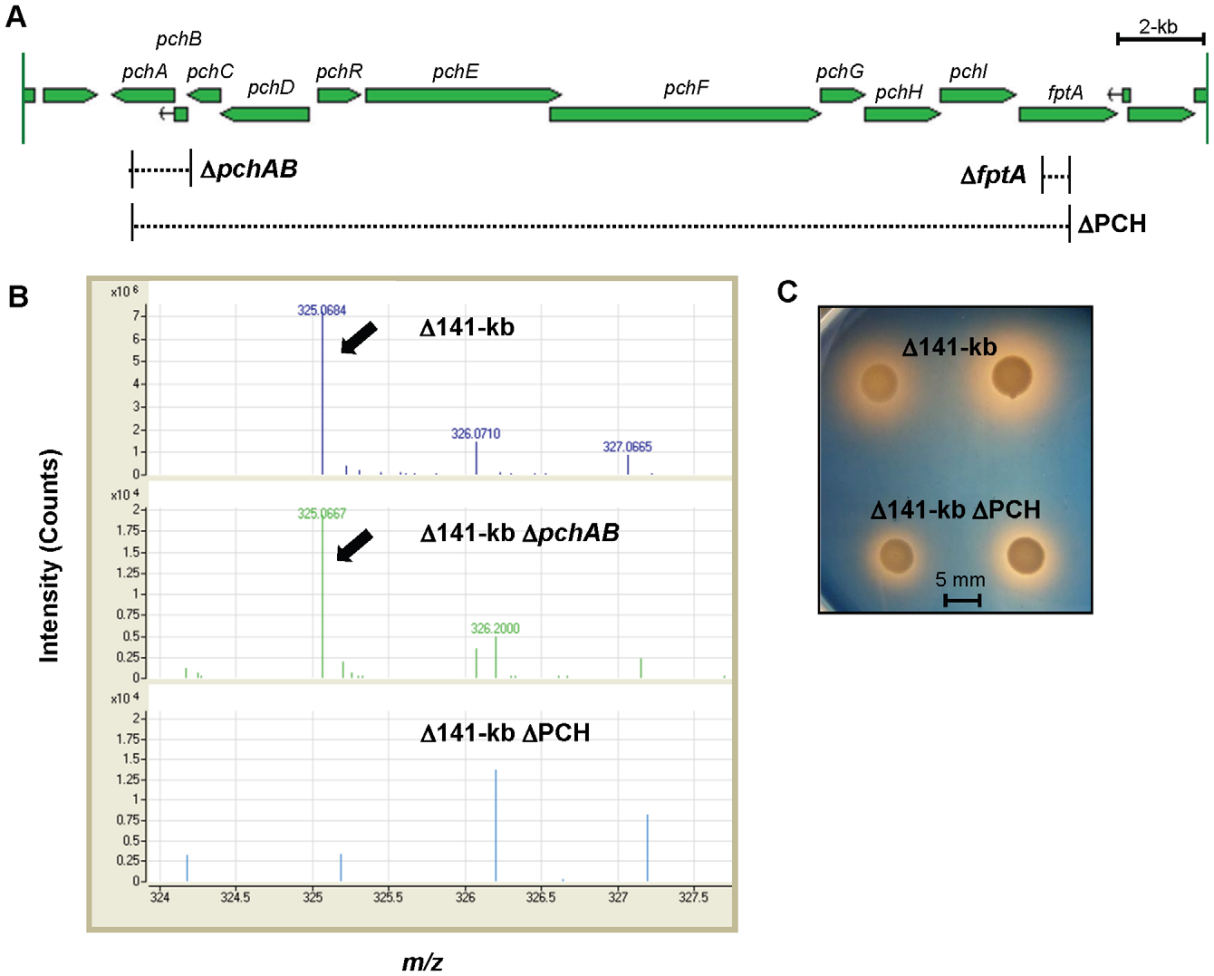
A *B. pseudomallei* 1710b malleobactin and pyochelin deficient double mutant exhibits siderophore activity. **A.** *Burkholderia* GBrowse map of the *B. pseudomallei* 1710b pyochelin synthesis and uptake gene cluster [62]. Gene names are labeled. Dashed lines bordered by vertical solid black lines indicate the extents of deletions. **B.** Detection of apo-pyochelin in the culture supernatant of pyochelin synthesis gene cluster mutants by mass spectrometry. TSBFC overnight cultures were filtered through 0.2 µM membranes, acidified and extracted with ethyl acetate. Extracts were dried under N_2_ and suspended in methanol. 5 µl samples were injected to detect the 325.068 M+H apo-pyochelin ion. The apo-pyochelin ion is indicated with arrows and isotope distribution ions are labeled. Note the different intensity scales in the top and bottom panels. **C.** To illustrate the presence of residual siderophore activity in Δ141-kb ΔPCH mutants, 5 µl samples from iron-limited TSBFC overnight cultures were spotted onto CAS agar plates and incubated at 37°C for 4 days prior to photographing.

The consistency of non-malleobactin halo production by 708a and 1710b malleobactin defective mutants ΔMBA and Δ141-kb caused us to speculate that expression of the known secondary siderophore pyochelin might be altered in 708a and other malleobactin defective mutants. To assess possible pyochelin contributions, several mutants defective in either pyochelin transport or synthesis (**Figure 2A**) were created in the Δ141-kb (Bp338) background by allelic exchange. These manipulations resulted in the following strains: Δ141-kb Δ*fptA* (Bp416), Δ141-kb Δ*pchAB* (Bp487), and Δ141-kb ΔPCH (Δ[*pchA-fptA*])(Bp516). These mutants are either defective in malleobactin (Δ141-kb) and/or pyochelin synthesis (Δ*pchAB*) or transport (Δ*fptA*) or both pyochelin synthesis and transport (ΔPCH).

Pyochelin production in parent and mutant strains was assessed by mass spectrophotometric analysis of products found in ethyl acetate-extracted cell free supernatants of TSBFC-grown cells. When compared to Δ141-kb, its Δ*pchA* and Δ*pchAB* mutant derivatives surprisingly still contained detectable, albeit reduced, traces corresponding to the 325.078 M+H ion of apo-pyochelin [44](**Figure 2B**), indicating that *B. pseudomallei* can produce salicylate independent of the presence of PchA and PchB. Apo-pyochelin was no longer detectable in supernatants obtained from ΔPCH mutant. Strain Δ141-kb ΔPCH lacking the malleobactin and pyochelin synthesis genes still produced halos on CAS plates indicating the probable presence of other siderophores (**Figure 2C**).

### Production of other siderophores

Because ΔPCH mutants still produce halos on CAS plates we analyzed supernatants for the presence of other possible siderophores. The analyses indicated that the ions corresponding to additional siderophores known to be produced by other *Burkholderia* species, e.g. cepabactin and cepaciachelin [21], [45]–[48], were undetectable by mass spectrometry. Mass spectrophotometric analysis of supernatants from the Δ141-kb and Δ141-kb ΔPCH mutants did, however, reveal a weak trace matching the 139.039 M+H ion expected for salicylate [49]. *B. pseudomallei* 1710b can therefore produce salicylate in the absence of PchA and PchB, the first two enzymes in the pyochelin biosynthetic pathway which produce salicylate from chorismate [50]. Use of salicylate as a siderophore has been reported for instance for *B. cenocepacia* [51]. In sufficient concentrations citrate can function as a siderophore [52]. To determine if the siderophore signal in the Δ141-kb ΔPCH strain was caused by citrate production we determined the citrate concentrations in uninoculated TSBFC and cell-free supernatants from TSBFC inoculated with Δ141-kb ΔPCH. Citrate concentrations in uninoculated TSBFC, which produces no CAS siderophore signal, were higher when compared to supernatants from TSBFC inoculated with Δ141-kb ΔPCH which does produce a CAS siderophore signal. This finding supports the notion that the halos produced by malleobactin and pyochelin deficient strains are not due to citrate and that *B. pseudomallei* may produce an unknown and uncharacterized molecule with iron chelating activity detected by the CAS ssay.

### Hemin and hemoglobin utilization requires the HMU locus but cannot be complemented by the HEM system

**Figure 3:**
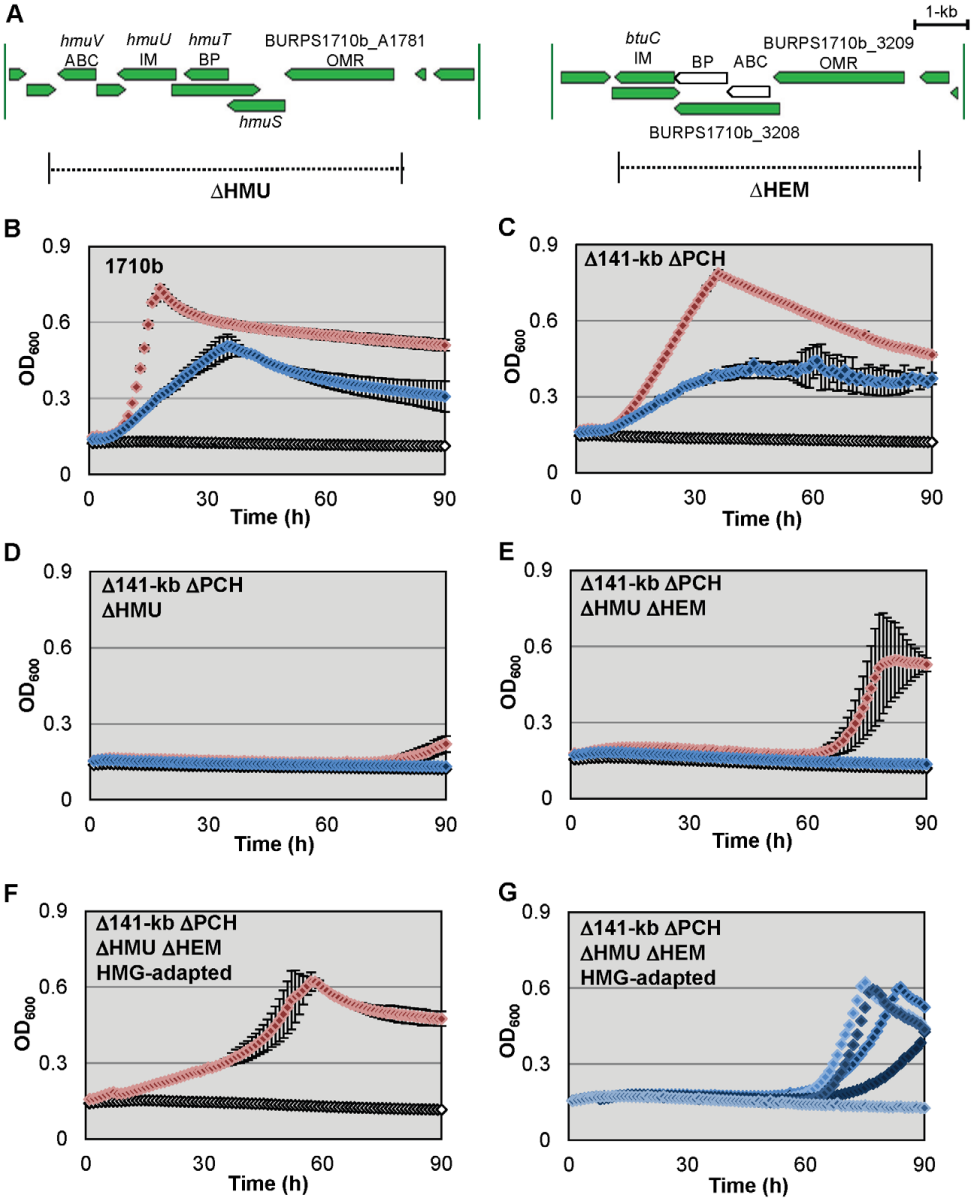
*B. pseudomallei* 1710b hemin utilization mutants exhibit hemin and hemoglobin growth defects that can be overcome by strain adaptation. **A.** *Burkholderia* GBrowse map of the *B. pseudomallei* 1710b annotated ABC transporter hemin uptake gene clusters [62]. In our revised annotation newly predicted open reading frames in the HEM locus appear as white open arrows. Gene names or locus numbers are listed. Predicted gene product functions are listed below gene names [29]. ABC; ATP-binding cassette subunit; IM, inner membrane protein; BP, periplasmic binding protein; OMR, TonB-dependent outer membrane receptor. Dashed lines bordered by vertical solid black lines and labeled by shorthand mutant nomenclature indicate the extents of deletions present in *hmu* and *hem* mutants. **B.–G.** *B. pseudomallei* 1710b (**B.**) and its *hmu* and *hem* mutant derivatives hemoglobin and hemin growth phenotypes (**C.** Δ141-kb ΔPCH, **D.** Δ141-kb ΔPCH ΔHMU, **E**. Δ141-kb ΔPCH ΔHMU ΔHEM, **F.** and **G.** Δ141-kb ΔPCH ΔHMU ΔHEM HMG-adapted). Microtiter plates containing 200 µl of M9-glucose minimal medium with 200 µM 2,2′-dipyridyl (open white symbols), or 200 µM 2,2′-dipyridyl and either 10 µM hemin (blue symbols) or 2.5 µM hemoglobin (red symbols) were inoculated with the indicated strains (in panel **F** only hemoglobin supplementation growth is shown and in panel **G** only hemin supplementation growth is shown). Cultures were incubated at 37°C with continuous aeration. The optical density at 600 nm (OD_600_) was measured hourly. OD_600_ means and standard deviation of three cultures from a single experiment are shown for all strains except for panel **G.** Six individual growth curves for Δ141-kb ΔPCH ΔHMU ΔHEM HMG-adapted in M9-glucose minimal medium with 200 µM 2,2′-dipyridyl and 10 µM hemin.

Microarray studies with cells grown in low-iron media indicated upregulation of *B. pseudomallei* hemin uptake genes in addition to malleobactin and pyochelin synthesis genes [28]. *B. pseudomallei* encodes two gene loci annotated for ABC transporter-dependent uptake of hemin (**Figure 3A**) [29]. The first hemin uptake locus (HMU) is located on chromosome II and is similar to the hemin uptake locus of *Yersinia spp*. The HMU system is a TonB-dependent ABC transporter which allows the use hemin or hemoproteins for growth under iron depleted conditions. The second hemin uptake locus, which we have called the HEM locus, is located on chromosome I. It contains *B. pseudomallei* K96243 homologs that have been re-annotated as likely components involved in hemin uptake [29]. Although it initially appeared that the 1710b HEM locus lacked a homolog of BPSL2723, which is predicted serve as the ATPase component of the transport system, closer examination revealed that this was due to a misannotated start site for BURPS1710b_3208, the predicted periplasmic binding protein. Based on homology with K96243 genes the correct annotation for BURPS1710b_3208 is from 3,515,337 bp to 3,516,380 bp which shortens its predicted open reading frame by almost half. An uncalled open reading frame with 99% sequence identity to BPSL2723 is located from 3,516,374 bp to 3,517,171 bp, immediately adjacent to the re-annotated BURPS1710b_3208 (**Fig. 3A**). Based on these observations the HEM locus appears to encode a functional ABC transporter in 1710b.

Strain 1710b and iron uptake mutants were tested for their ability to use hemin or hemoglobin for growth in iron-depleted M9-glucose medium (**Figure 3B–G**). 1710b and Δ141-kb ΔPCH grew readily in iron-depleted media when hemin or hemoglobin were provided at a heme iron concentration of 10 µM (**Figure 3B and C**). However, neither the Δ141-kb ΔPCH ΔHMU nor the Δ141-kb ΔPCH ΔHMU ΔHEM strain could readily utilize hemin or hemoglobin as an iron source (**Figure 3D and E**). The inability of the HEM locus present in Δ141-kb ΔPCH ΔHMU to compensate for the loss of the HMU locus implies that the HEM locus may not be involved in the utilization of hemin in 1710b.

Interestingly, while growth with hemin supplementation was not observed in ΔHMU strains in freshly inoculated cultures, both the Δ141-kb ΔPCH ΔHMU and Δ141-kb ΔPCH ΔHMU ΔHEM strains adapted to utilize hemoglobin when cultures were incubated for several days (**Figure 3D and E**). For Δ141-kb ΔPCH ΔHMU ΔHEM this hemoglobin adaptation effect occurred reliably after about 60 h of incubation in nearly every experiment (**Figure 3E**). Δ141-kb ΔPCH ΔHMU also appeared to have the capacity to adapt to hemoglobin utilization, although it occurred much later in the incubation periods, typically near the end of day four (**Figure 3D**). When Δ141-kb ΔPCH ΔHMU ΔHEM cells were recovered from hemoglobin adapted cultures and single colony purified, the hemoglobin adapted strain grew with hemoglobin as an iron source without an appreciable lag phase, albeit at a slower rate than HMU^+^ strains (**Figure 3F**). Additionally, hemoglobin adapted Δ141-kb ΔPCH ΔHMU ΔHEM derivatives were subsequently able to sporadically adapt to hemin utilization after a two to three day lag (**Figure 3G**). This hemin adaptation did not occur in every trial or even in every subculture inoculated in triplicate with cells from the same Δ141-kb ΔPCH ΔHMU ΔHEM starter culture.

### Lactoferrin but not ferritin-iron utilization is dependent on malleobactin synthesis

**Figure 4:**
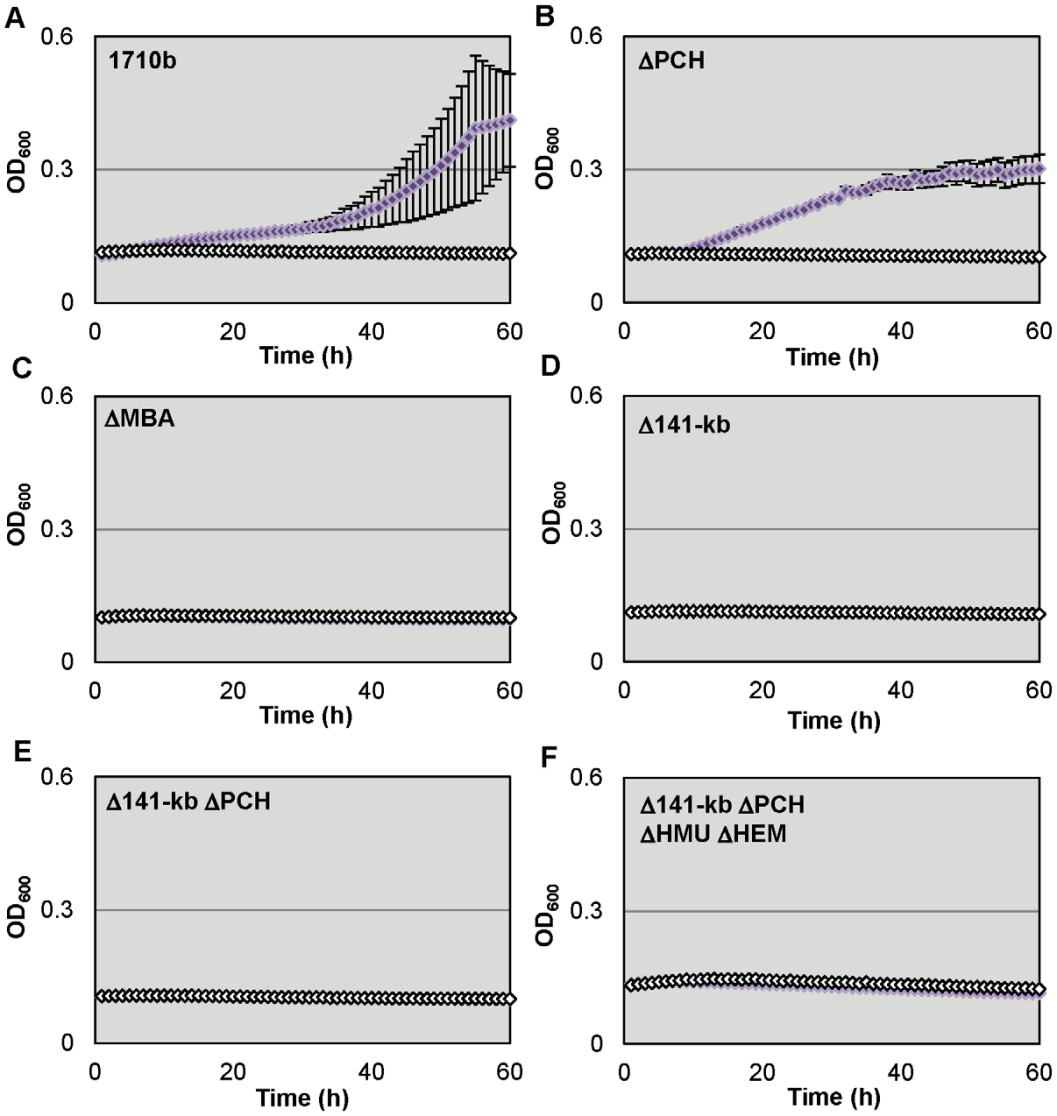
*B. pseudomallei* 1710b malleobactin mutants cannot use lactoferrin as an iron source A.–F. Microtiter plates containing 200 µl of M9-glucose minimal medium with 200 µM 2,2′-dipyridyl (open white symbols), or 200 µM 2,2′-dipyridyl and 1 µM lactoferrin (purple symbols) were inoculated with strain 1710b (**A.**) and its siderophore synthesis and hemin uptake mutants (**B.** ΔPCH, **C.** ΔMBA, **D.** Δ141-kb, **E.** Δ141-kb ΔPCH and **F.** Δ141-kb ΔPCH ΔHMU ΔHEM). The optical density at 600 nm (OD_600_) was measured hourly. OD_600_ means and standard deviation of three cultures from a single experiment are shown for all strains.

We also tested strain 1710b and and its siderophore synthesis and hemin uptake mutants (ΔPCH, ΔMBA, Δ141-kb, Δ141-kb ΔPCH and Δ141-kb ΔPCH ΔHMU ΔHEM) for their ability to utilize lactoferrin as iron sources (**Figure 4**). Although studies with lactoferrin are complicated by its low solubility, deferrated M9-glucose medium containing 1 µM lactoferrin supported growth of 1710b (**Figure 4A**) and ΔPCH (**Figure 4B**). In contrast, growth of ΔMBA, Δ141-kb, Δ141-kb ΔPCH and Δ141-kb ΔPCH ΔHMU ΔHEM was not supported at the lactoferrin concentration tested (**Figure 4C–F**). Thus, under the experimental conditions employed here lactoferrin utilization is only dependent on malleobactin production.

**Figure 5:**
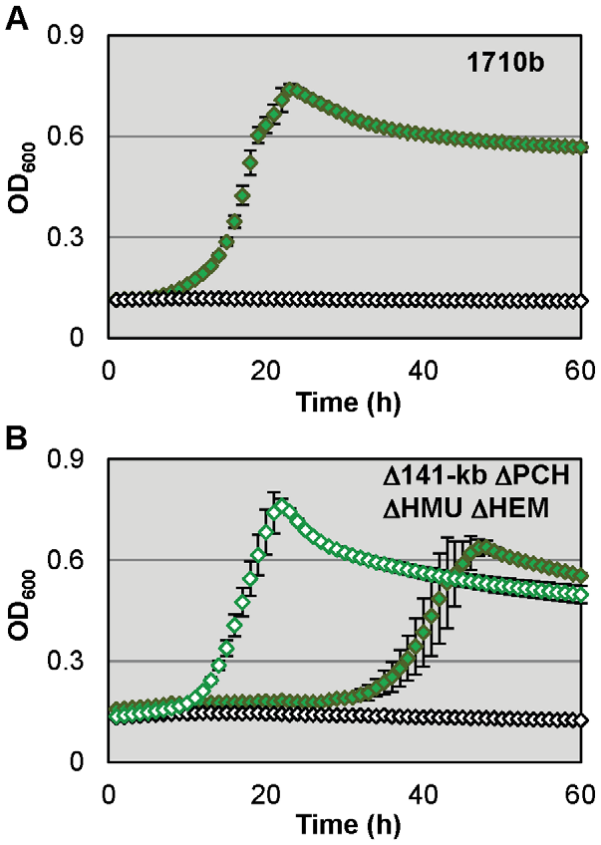
*B. pseudomallei* 1710b siderophore synthesis and hemin uptake mutant can utililize ferritin as an iron source. **A.** Microtiter plates containing 200 µl of M9-glucose minimal medium with 200 µM 2,2′-dipyridyl (open white symbols), or 200 µM 2,2′-dipyridyl and 10 µg/ml ferritin (green symbols) were inoculated with strain 1710b. The optical density at 600 nm (OD_600_) was measured hourly. OD_600_ means and standard deviation of three cultures from a single experiment are shown for all strains **B**. Δ141-kb ΔPCH ΔHMU ΔHEM was grown in the same media and using the same conditions as described above in **A** for 1710b, except that two different 2,2′-dipyridyl concentrations were used: 100 µM (open green symbols and open white symbols) and 200 µM (closed green symbols).

Strains 1710b and Δ141-kb ΔPCH ΔHMU ΔHEM were both able to readily utilize ferritin as an iron source at a concentration of 10 µg/ml (**Figure 5A and B**). Ferritin-iron dependent growth kinetics were affected by chelator concentrations. At 200 µM 2,2′-dipyridyl which was required for complete growth suppression of 1710b in M9-glucose medium without added iron source, Δ141-kb ΔPCH ΔHMU ΔHEM growth was delayed compared to 1710b. However, when this strain was grown in the presence of 100 µM 2,2′-dipyridyl which was sufficient for complete growth suppression of Δ141-kb ΔPCH ΔHMU ΔHEM without added iron source, 1710b and Δ141-kb ΔPCH ΔHMU ΔHEM growth kinetics were similar. Of all the host iron sources studied, ferritin was the only one that allowed similar growth of 1710b and Δ141-kb ΔPCH ΔHMU ΔHEM. The utilization of ferritin-bound iron has been described previously only in one other pathogen, namely *B. cenocepacia* [22].

### Lethality in the acute murine melioidosis model does not require siderophore production and hemin uptake systems

**Figure 6:**
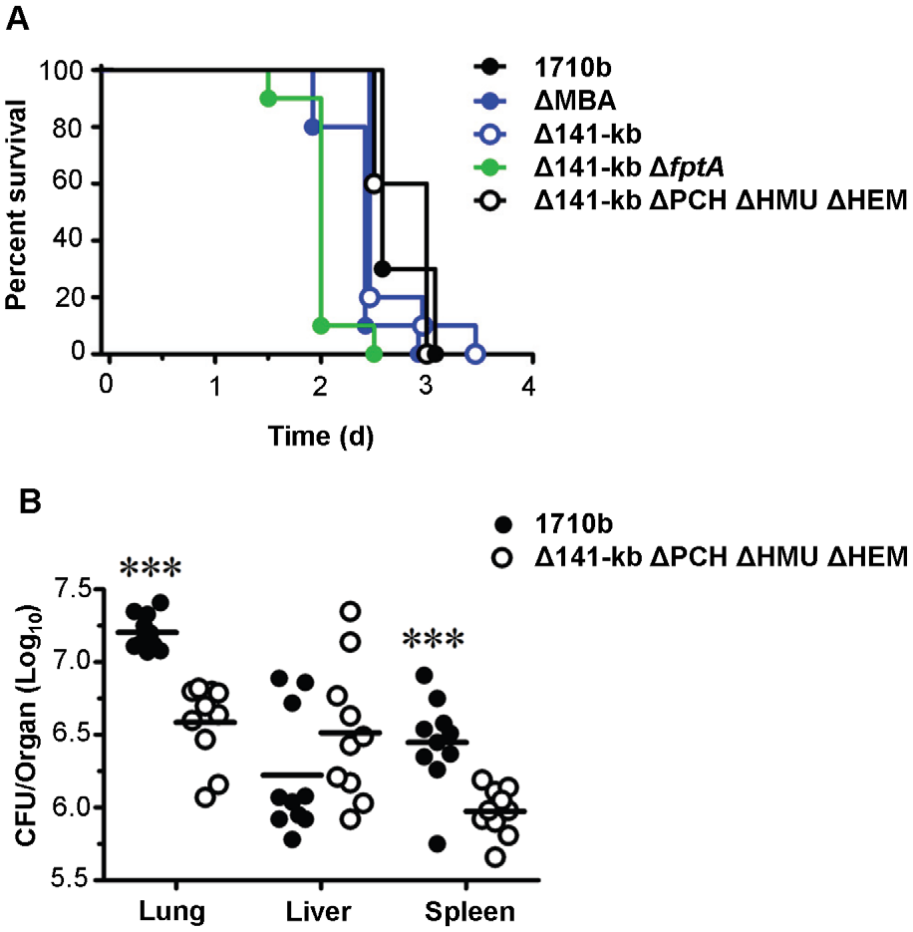
Lethality of strain 1710b and its various iron acquisition mutants. **A.** Kaplan-Maier survival curves of BALB/c mice infected intranasally with 1710b (3×10^3^ CFU), ΔMBA (3.5×10^3^ CFU), Δ141-kb (2.1×10^3^ CFU), Δ141-kb Δ*fptA* (1.9×10^3^ CFU), or Δ141-kb ΔPCH ΔHMU ΔHEM (3.8×10^3^ CFU). Survival was monitored and mice were euthanized upon reaching a pre-determined endpoint. Data were pooled from two independent experiments (total n = 10 per bacterial strain). For comparison of each mutant to 1710b the Bonferroni correction for multiple comparisons was applied. p values were as follows: ΔMBA, p = 0.12; Δ141-kb, p = 0.93; Δ141-kb Δ*fptA*, p = 0.0001; Δ141-kb ΔPCH ΔHMU ΔHEM, p = 0.19. **B.** Organ bacterial burden from endpoint mice (days 2.5 and 3) following intranasal challenge with 1710b or Δ141-kb ΔPCH ΔHMU ΔHEM. BALB/c mice (n = 10 per bacterial strain) were infected intranasally with 1710b (3×10^3^ CFU) or Δ141-kb ΔPCH ΔHMU ΔHEM (3.8×10^3^ CFU). Bacterial burdens from mice were determined in lung, liver and spleen using data pooled from mice euthanized at endpoint (days 2.5 and 3) as described in Materials and Methods. Significant differences between 1710b and Δ141-kb ΔPCH ΔHMU ΔHEM were determined by a two-tailed Student's t-test (*** = p<0.0001). Data are graphed as individual values, with bars representing the mean log_10_ CFU/organ titer for each group. Data were pooled from two independent experiments.

The lethality of 1710b and four of the 1710b-derived siderophore and hemin utilization mutants was tested in an acute intranasal (i.n.) challenge murine melioidosis model. BALB/c mice received a lethal i.n. challenge dose of LB-grown∼3×10^3^ CFU of either strain 1710b or the mutants generated in this study which included ΔMBA, Δ141-kb, Δ141-kb Δ*ftpA*, and Δ141-kb ΔPCH ΔHMU ΔHEM. Deletion of none of these genes or gene clusters significantly attenuated the lethality in the murine melioidosis model, with the quadruple Δ141-kb ΔPCH ΔHMU ΔHEMmutant showing a virtually indistinguishable survival curve when compared to the parental strain 1710b (**Figure 6A**). The types and timing of clinical symptoms development was similar following infection with individual strains and mice reached euthanasia endpoints at similar times (2.5 to 3.5 days). Although time-to-death was not significantly attenuated in the examined strains, dissemination was affected. Organ burdens with the quadruple iron acquisition mutant were significantly lower in the lung and spleen, but unchanged in the liver (**Figure 6B**). While we do not yet understand the differences in organ burdens with the various strains, it is interesting to note that Δ141-kb ΔPCH ΔHMU ΔHEM trends to multiply better in the liver, the primary organ for iron storage.

## Discussion

For several pathogens the loss of their primary high-affinity siderophore systems results in a significant attenuation in virulence [15]–[20]. Malleobactin had been previously shown to be capable of acquiring iron from transferrin or lactoferrin and it had been assumed to act as an important virulence factor in melioidosis [24]. Therefore the absence of the malleobactin synthesis genes in the clinical isolate 708a came as a surprise as the strain remained fully virulent in the acute murine melioidosis model [32]. This is in contrast to *Galleria mellonella* wax moth caterpillar model in which 708a was attenuated [53], [54]. Loss of malleobactin production could possibly represent a beneficial pathogenic adaptation by 708a in response to some environmental cue. During infection *B. pseudomallei* rapidly deletes large portions of its chromosomal DNA in response to adverse conditions. For example, ceftazidime treatment of acute *B. pseudomallei* infections leads to emergence of resistant isolates with large chromosomal deletions removing the penicillin binding protein target within a few weeks after therapeutic intervention [54]. Deletion of siderophore biosynthesis genes has been observed in *P. aeruginosa* which frequently loses the ability to synthesize pyoverdin during chronic cystic fibrosis infections [55].

The absence of the malleobactin synthesis genes from *B. pseudomallei* 708a argues against malleobactin being required for this bacterium's virulence in the murine melioidosis acute infection model. However, given *B. pseudomallei*'s ability to rapidly adapt to adverse conditions within the host which may have endowed strain 708a to acquire iron in the absence of malleobactin, for instance by upregulating the production of other siderophores or iron-acquisition mechanisms. We therefore decided to investigate the role of annotated, but not yet characterized, iron acquisition systems in *B. pseudomallei*'s biology and virulence in a defined genetic background. To this end, we systematically deleted the malleobactin, pyochelin and hemin utilization systems and determined their requirements for *in vitro* growth on various iron sources, as well as lethality in the murine meliodosis model.

Recreation of the 708a 141-kb deletion or engineering of a Δ(*mbaS-mbaF*) deletion removing the malleobactin synthesis genes resulted in mutants with reduced siderophore levels within a 24 h period as assessed by qualitative and quantitative CAS assays, presumably due to lack of malleobactin production (**Figure 1B and C**). However, after prolonged (4 d) incubation the 1710b derived mutant strains lacking the *mba* gene cluster produced siderophore phenotypes which quantitatively and qualitatively were almost identical to those observed with 708a (**Figure 1C and D**). Malleobactin mutant derivatives of 1710b retained full lethality in the acute murine intranasal challenge melioidosis model supporting the notion that this siderophore is not a major *B. pseudomallei* virulence determinant. However, in the current study all strains were grown in iron-replete LB medium which may affect the degree of lethality/virulence observed in our challenge model. Additionally, the current study does not address how iron-acquisition mutants would possibly behave in a chronic *B. pseudomallei* infection model. Collectively, we interpret these results to mean that 708a does not carry mutations that constitutively up-regulate secondary siderophore production, but rather that absence of malleobactin may lead to induction of and/or shift to utilization of alternate iron-acquisition pathways that compensate for the loss of malleobactin.

*B. pseudomallei* had been previously confirmed to produce the secondary siderophore pyochelin [25]. PchA (isochorismate synthase) and PchB (isochorismate pyruvate-lyase) catalyze the initial two steps in the pyochelin synthetic pathway by converting chorismate to salicylate [50]. Surprisingly, *pchA* and *pchB* mutants still produced apo-pyochelin whose synthesis was completely abrogated in a mutant where the entire *pch* operon was deleted. However, salicylate could still be detected in this background indicating that *B. pseudomallei* possesses an additional pathway for salicylate synthesis which was able to feed into the pyochelin biosynthetic pathway in the absence of PchA and PchB. While *Pseudomonas* and *Burkholderia* sp. produce pyochelin, its contribution to virulence is typically minor [56]. Pyochelin overproducing strains of *B. cenocepacia* could not compensate for virulence defects caused by the loss of ornibactin [57]. Consistent with data obtained with other pyochelin-producing pathogens this siderophore also seems to play a minor role in *B. pseudomallei* pathogenesis as the pyochelin synthesis mutant ΔPCH exhibited no discernible effects on lethality in the acute murine melioidosis model when compared to strains (1710b, ΔMBA and Δ141-kb) that still produced pyochelin (**Figure 6A**). Interestingly, when compared to 1710b containing an intact TonB-dependent FptA pyochelin receptor, the Δ141-kb Δ*fptA* mutant exhibited a statistically significant (p = 0.0001) increase in lethality in this infection model. While this difference was statistically significant, the biological relevance of this observation is difficult to assess since mean survival of Δ141-kb Δ*fptA* was reduced by less than one day (2.0 days for Δ141-kb Δ*fptA* versus 2.7 days for 1710b).

The 1710b malleobactin and pyochelin deficient strains still produced significant halos on CAS plates (**Figure 3C**). We determined that this iron-chelating activity was not due the presence of citrate which can act as a siderophore at sufficiently high concentrations [52]. Additionally, other known *Burkholderia* siderophores, cepabactin and cepaciachelin [21], [45]–[48], were undetectable by mass spectrometry. *B. pseudomallei* may therefore produce a yet uncharacterized siderophore. ΔPCH mutants produced salicylate which has been shown to function as a siderophore in *B. cenocepacia* [51]. However, the role of salicylate as a siderophore remains controversial [21]. Without additional experimentation, we cannot eliminate the possibility that *B. pseudomallei* may induce the salicylate pathway in absence of other siderophores.

Siderophores are commonly synthesized by non-ribosomal peptide synthetase/polyketide synthase (NRPS/PKS) gene clusters. *B. pseudomallei* K96243 encodes 12 NRPS/PKS gene clusters besides the two clusters used to synthesize malleobactin and pyochelin [26]. None of these NRPS/PKS gene clusters were observed to be directly upregulated in microarray analysis of RNA from cells grown under conditions of limiting iron [28]. It is possible that siderophore synthesis gene expression is hierarchical and the genes for synthesis of the unknown siderophore activity may not be upregulated if malleobactin and pyochelin are present. However BPSS0312, a LuxR-type regulator immediately downstream of the BPSS0299–BPSS0311 NPRS/PKS cluster was seen to be upregulated under low iron conditions [28]. This NPRS/PKS cluster is linked to transport genes and is conserved in *B. mallei* [26]. It is also possible that the siderophore activity observed in ΔMBA and ΔPCH mutants is synthesized by a NRPS-independent mechanism. We presently cannot rule out a role of this uncharacterized siderophore in pathogenicity.

To our knowledge hemin uptake in *B. pseudomallei* has not been previously been characterized. Loss of heme utilization is not associated with virulence defects in many of the organisms in which it has been studied, but typically these studies looked at heme utilization alone rather than in combination with siderophores [58], [59]. Here we confirm that the *B. pseudomallei* HMU locus is involved in hemin utilization under low iron conditions (**Fig. 3D**). However, the HEM locus appears to be unrelated to hemin utilization in 1710b based on its failure to compensate for the loss of the HMU locus. Although the HEM locus might be involved in hemin utilization in other strains of *B. pseudomallei* such as K96243, the *hem* genes were not up-regulated in K96243 under low iron conditions [28]. In addition, *B. pseudomallei* has an uncharacterized secondary capacity to utilize heme sources in the absence of the HMU and HEM loci as demonstrated by the hemoglobin adaptation phenotype (**Figure 3E and F**). We believe that the reliability and consistency in the emergence timing of the hemoglobin adaptation phenotype supports the notion that this adaptation could be a controlled genetic event. In contrast, the unreliability of the subsequent hemin adaptation phenotype may indicate that it is due to a commonly occurring mutation.

Analysis of the ability of the mutants to utilize host iron sources other than hemin and hemoglobin revealed that 1710b could readily utilize lactoferrin-derived iron and this required malleobactin but not pyochelin or other siderophore production. In contrast, 1710b and Δ141-kb ΔPCH ΔHMU ΔHEM could use ferritin as iron source equally well. Ferritin-iron acquisition has previously been demonstrated in *B. cenocepacia* and this mechanism seems to require proteolytic ferritin degradation by a serine protease [22]. However, to our knowledge the ferritin-iron acquisition pathway has not yet been characterized in more detail and its role in virulence not yet been assessed.

The quadruple Δ141-kb ΔPCH ΔHMU ΔHEM mutant strain is unable to produce malleobactin, pyochelin and hemin acquisition systems, yet displayed no significant reduction in lethality based on survival in an acute murine melioidosis model (**Figure 6B**). This indicates that each of these systems is dispensable for lethality both individually and in combination. When compared to 1710b, bacterial loads were slightly, but significantly decreased in the lung and spleen, but not the liver. Ready dissemination from the site of infection and lack of a load difference in the liver argues against any general defect in *in vivo* replication. The lung and spleen load reductions may be associated with a slight *in vivo* iron uptake defect of the quadruple mutant strain that is alleviated by iron stores in the liver.

The lack of *B. pseudomallei* lethality phenotypes associated with siderophores may be associated with its intracellular lifestyle as many of the bacterial pathogens with strong siderophore-based virulence defects are extracellular pathogens. For instance, *a Brucella abortus* brucebactin deficient strain grew similarly to a wild-type strain in a mouse macrophage model [60]. Intracellular bacteria have access to iron sources such as ferritin or may live in microaerophilic or anaerobic environments where iron is available as Fe^2+^ and can thus be acquired by siderophore-independent mechanisms [61]. Ferritin utilization may explain the unchanged lethality of the mutant strains, but the specific strategy used by *B. pseudomallei* to acquire host iron remains unclear.

Based on our results obtained thus far we favor a model by which *B. pseudomallei* liberates iron from ferritin, possibly using a secreted protease as previously demonstrated in *B. cenocepacia* [22]. Other players involved in ferritin-iron acquisition possibly include ferric or ferrous iron-transport systems, iron reductases, or heme oxygenases. Only a detailed genetic analysis of the ferritin-iron acquisition pathway will reveal the players involved in this process and shed light on the importance of this pathway in *B. pseudomallei*'s pathogenicity. The work presented here provides the framework for dissection of iron-acquisition pathways required for *in vivo* survival of this highly versatile and adaptable pathogen.

## Supporting Information

Table S1 **Oligonucleotides used in this study.**
(DOC)

Table S2 **Plasmids used in this study.**
(DOC)

Text S1 (DOC)
