# Supplementary material for: Burkholderia pseudomallei Known Siderophores and Hemin Uptake Are Dispensable for Lethal Murine Melioidosis
Source: PLoS Negl Trop Dis. 2012 Jun 26;6(6):e1715. doi: 10.1371/journal.pntd.0001715 (PMC3383733; doi:10.1371/journal.pntd.0001715)
Supplement: Table S1 — Oligonucleotides used in this study. (DOC) [file pntd.0001715.s001.doc]

**Table S1. Oligonucleotides used in this study**

| **P stock #** | **Name** | **Sequence 5’→3’** |
| --- | --- | --- |
| 536 | oriT-UP | Tccgctgcataaccctgcttc |
| 537 | oriT-DN | Cagcctcgcagagcaggattc |
| 1597 | Km-UP-pCR2.1 | Taacaaatgcatggcgcaaggg |
| 1655 | npt Probe Up | aatatcacgggtagccaacg |
| 1656 | npt Probe Dn | GCCCTGAATGAACTCCAAGA |
| 1963 | Hind-psbSupreg-rev | TAATAAGCTTGGATTCTCCAGATGTTTG |
| 1968 | Eco-psbSupreg-For.2 | taagaattccgatcctgttcctgtcgag |
| 1970 | Hind-mbaFdwnreg-for.2 | tataagcttggctccgctacacatggat |
| 1971 | Eco-mbaFdwnreg-rev.2 | taagaattcgcgcacgaaacagtagatga |
| 2069 | XhoI-RBS-fptA-for.2 | CTCGAGGGGATTTATGCGGTACGGG |
| 2070 | fptA-rev.2 | ATAGCGGATCGTGTCGAACGTGTAG |
| 2079 | pchA-L-for | AACCGATCGAACCGGACGAC |
| 2080 | HinDIII-pchA-L-rev | AAGCTTAACGATCCGGCTCACGAT |
| 2081 | HindIII-pchA-R-for | AAGCTTACCAGCAGTTTCGCAAGCGTCAT |
| 2082 | XhoI-pchA-R-rev | Ctcgag ACAGCAAACGCGGCACT |
| 2090 | DfptA.chk.for | GCTGAAGGCGATTTCGGTGA |
| 2091 | DfptA.chk.rev | TGCGAGATGTCGTTCTGCATGT |
| 2130 | pchBA-rev | tcgatgcctcgtcagtgc |
| 2152 | HindIII-pchC-for | AAGCTTaTTGGTCCGGTGTCTTCATGAGTGT |
| 2153 | pchC-rev | CATCGGCAAGACCAGCAAGAAATCGCT |
| 2212 | DpchBA.chk.for.1 | TCGTGGTGCCGGATGTCGT |
| 2213 | FK.chk.rev | AGCGCTCTGAAGTTCCTATACTTTCT |
| 2214 | DpchA.chk.for | TCTTTCGCCGACGCCATCA |
| 2215 | hind-fptA-for | aagcttATTTCGACTGGGATACGACGC |
| 2273 | Eco-DHMU-Lflnk-for | CACCGAATTCCGACGGCGATTTCGTCTATG |
| 2274 | Hind-DHMU-Lflnk-rev.2 | TAATAAGCTTATCCTGAGCGCGAATCCCA |
| 2275 | Hind-DHUM-Rflnk-for | TAATAAGCTTTTGGTCTCCCAGTGACGTTT |
| 2276 | Eco-DHMU-Rflnk-rev | AC GAATTCGACCGGCAAATCCAGC |
| 2295 | pFKM2-QC-PacI | CAATGGAATAATTAATTAAGTGATtGGAAATGTTGg |
| 2296 | DHMU.chk.F | TGTATGTCGGCATGAAGGCGAT |
| 2297 | DHMU.chk.R | TCAGGAGTTGCTGTTCGTCTCGTT |
| 2300 | TOPO-Hind-DHEMR-F | CACCAAGCTTCGCACGCGGCTGGA |
| 2301 | Eco-DHEMR-R | TAATGAATTCGTGATGCGTGACGGGCGATATG |
| 2304 | TOPO-ECO-DHEML-F | CACCGAATTC AATCGAGCGAAGTCAGGCGCATC |
| 2305 | Hind-DHEML-R.2 | TAATAAGCTTTTCGCACGGCGAATCGAGG |
| 2331 | DHEM.chk.F | tgccgatggtgctctattgcg |
| 2332 | DHEM.chk.R | ttccagcaggtgagcgagctgtt |
