# Supplementary material for: Burkholderia pseudomallei Known Siderophores and Hemin Uptake Are Dispensable for Lethal Murine Melioidosis
Source: PLoS Negl Trop Dis. 2012 Jun 26;6(6):e1715. doi: 10.1371/journal.pntd.0001715 (PMC3383733; doi:10.1371/journal.pntd.0001715)
Supplement: Table S2 — Plasmids used in this study. (DOC) [file pntd.0001715.s002.doc]

**Table S2. Plasmids used in this study**

| **Plasmid** | **Relevant features** | **GenBank accession number** | **Source or Reference** |
| --- | --- | --- | --- |
| pEXKm4 | Allelic exchange vector;  Km^R^; *sacB* (Suc^S^); I-SceI counterselection | FJ797516 |  |
| pEXKm5 | Allelic exchange vector;  Km^R^; *sacB* (Suc^S^); I-SceI counterselection; allows blue-white selection of merodioplids due to *uidA* gene | GQ200735 |  |
| pEXGm5B | Allelic exchange vector;  Gm^R^; *sacB* (Suc^S^); I-SceI counterselection; allows blue-white selection of merodioplids due to *uidA* gene |  | This study |
| pCR2.1 | TA-cloning vector; Amp^R^, Km^R^ |  | Invitrogen, Carlsbad, CA |
| pGEM®-T Easy | TA-cloning vector; Amp^R^ |  | Promega, Madison, WI |
| pBADSce | *Burkholderia* ts replicon; Zeo^R^;  arabinose inducible expression of I-*Sce*I | FJ797515 |  |
| pPS856 (pFGm) | Source of Gm^R^ gene |  |  |
| pFKm2 | *FRT* flanked Km^R^ cassette; Km^R^ | EU215433 |  |
| pFKm4 | *FRT* flanked Km^R^ cassette; Km^R^ |  | This study |
| pCC1FOS-708a-6 | pCC1FOS with 708a 141-kb deletion borders |  |  |
| pEXKm4ΔMBA::FRTKm | ∆(*mbaS-mbaF*)::*FRT*-*npt*II-*FRT* deletion construct |  | This study |
| pEXGm5Δ141::FRTKm | ∆(BURPS1710b_2054-BURPS1710b_2155)::*FRT*-*npt*II-*FRT* deletion construct |  | This study |
| pEXKm5Δ*fptA* | ∆*fptA* deletion construct |  | This study |
| pEXGm5Δ*pchA*::FRTKm | ∆*pchA*::*FRT*-*npt*II-*FRT* deletion construct |  | This study |
| pEXGm5Δ*pchBA*::FRTKm | ∆(*pchBA*)::*FRT*-*npt*II-*FRT* deletion construct |  | This study |
| pEXGm5ΔPCH::FRTKm | ∆(*pchA*-*fptA*)::*FRT-nptII-FRT* deletion construct |  | This study |
| pEXGm5ΔHMU::FRTKm | ∆(*hmuV-* BURPS1710b_A1781)::*FRT*-*npt*II-*FRT* deletion construct |  | This study |
| pEXGm5ΔHEM::FRTKm | *∆*(*btuC-*BURPS1710b_3209)::*FRT*-*npt*II-*FRT* deletion construct |  | This study |

Abbreviations: Amp, ampicillin; Gm, gentamicin; Km, kanamycin; R, resistance; S, sensitive/susceptible; Suc, sucrose; Zeo, zeocin.

**References**

1. Lopez CM, Rholl DA, Trunck LA, Schweizer HP (2009) Versatile dual-technology system for markerless allele replacement in *Burkholderia pseudomallei*. Appl Environ Microbiol 75: 6496-6503.

2. Hoang TT, Karkhoff-Schweizer RR, Kutchma AJ, Schweizer HP (1998) A broad-host-range Flp-*FRT* recombination system for site-specific excision of chromosomally-located DNA sequences: application for isolation of unmarked *Pseudomonas aeruginosa* mutants. Gene 212: 77-86.

3. Choi K-H, Mima T, Casart Y, Rholl D, Kumar A, et al. (2008) Genetic tools for select agent compliant manipulation of *Burkholderia pseudomallei*. Appl Env Microbiol 74: 1064-1075.

4. Trunck LA, Propst KL, Wuthiekanun V, Tuanyok A, Beckstrom-Sternberg SM, et al. (2009) Molecular basis of rare aminoglycoside susceptibility and pathogenesis of *Burkholderia pseudomallei* clinical isolates from Thailand. PLoS Negl Trop Dis 3: e0000519.
