# Supplementary material for: Burkholderia pseudomallei Known Siderophores and Hemin Uptake Are Dispensable for Lethal Murine Melioidosis
Source: PLoS Negl Trop Dis. 2012 Jun 26;6(6):e1715. doi: 10.1371/journal.pntd.0001715 (PMC3383733; doi:10.1371/journal.pntd.0001715)
Supplement: Text S1 — (DOC) [file pntd.0001715.s003.doc]

**Supplementary Materials**

**Strain Construction**

**Bp327.** DNA fragments corresponding to 1.15-kb upstream sequences of *mbaS* and 0.85-kb downstream sequences of *mbaF* were PCR-amplified from 1710b genomic DNA using primers 1968+1963 and 1970+1971 (oligonucleotides used in this study are listed in **Table S1**), respectively, and cloned into the TA-cloning vector pCR2.1 (plasmids used in this study are listed in **Table S2**). The upstream and downstream DNA fragments were released from the corresponding pCR2.1 constructs by digestion with *Hin*dIII+*Spe*I and *Hin*dIII+*Eco*RI, respectively, ligated with T4 DNA ligase, and re-digested with *Eco*RI. The resulting 2-kb product was cloned into the *Eco*RI site of pEXKm4. Next, the 1.5-kb *FRT*-*npt*II-*FRT* *Hin*dIII fragment from pFKM2 containing the Km^R^ marker was ligated into the central *Hin*dIII site between the upstream and downstream segments to create pEXKm4ΔMBA::FRTKm. This plasmid was conjugated into 1710b and Km^R^ merodiploids were selected. Transformation of a merodiploid isolate with pBADSce, followed by arabinose induction of I-*Sce*I counter-selection, selection of Km^R^ colonies, and pBADSce curing resulted in a ΔMBA::*FRT*-Km^R^-*FRT* mutant. The Km^R^ marker was then removed using Flp recombinase following a previously described protocol . The presence of the desired deletion in the resulting strain Bp327 was confirmed by Southern blot analysis using *Eco*RI-digested chromosomal DNA and the 1.15-kb upstream flanking DNA fragment as the probe.

**Bp338**. The 2.3-kb *Xho*I fragment carrying the 141-kb deletion junction from pCC1FOS-708a-6 was cloned into pWSK29 . pWSK29::Δ141 was digested with *Fse*I to remove a 0.5-kb central portion, blunt-ended with T4 polymerase and ligated with the 1.5-kb *FRT*-*npt*II-*FRT* *Sma*I fragment from pFKM2 containing the Km^R^ marker. The 3.3-kb marked deletion was sub-cloned on a *Xho*I fragment into pEXGm5B digested with the same enzyme to create pEXGm5Δ141::FRTKm. This plasmid was conjugally transferred into strain Bp74 and merodiploids were selected on Gm+Km+Zeo+X-gluc-containing plates. Sucrose counter-selection in the presence of kanamycin was performed to obtain a Δ141::FRTKm mutant. White colonies were then tested for Gm^S^, Km^R^ and Zeo^S^. The Km^R^ marker was then removed using Flp recombinase following a previously described protocol . The presence of the desired deletion in the resulting strain Bp338 was confirmed by Southern blot analysis using *Sca*I-digested chromosomal DNA and the 2.3-kb deletion junction-containing DNA fragment as the probe.

**Bp416.** A 1.9-kb fragment of *fptA* was PCR-amplified from 1710b genomic DNA with primers 2069 and 2070, and cloned into pGEM®-T Easy. The resulting pGEM®-T Easy*fptA* was then digested with *Bsi*WI to remove a 0.67-kb central DNA fragment, followed by religation*.* The 1.3-kb Δ*fptA* fragment was then rescued by *Eco*RI digestion and cloned into pEXKm5 to create pEXKm5Δ*fptA.* This plasmid was transferred to Bp338 by conjugation, merodiploid exconjugants were selected on Km-containing medium, followed by sucrose counter-selection to resolve the merodiploid and recover the Δ*fptA* mutant. White colonies were patched for Km^S^. The presence of the Δ*fptA* allele was confirmed by PCR-amplification of the deletion-containing region from Bp416 chromosomal DNA with primers 2090 and 2091, followed by sequencing using the same primers.

**Bp447, Bp448 and Bp449.** Two fragments of 0.75-kb and 0.5-kb corresponding to the 3’ and 5’ regions of *pchA* were PCR-amplified from 1710b genomic DNA with primers 2079+2080 and 2081+2082, respectively, and into pCR2.1. The DNA fragments corresponding to the left and right flanking regions were released from pCR2.1 by *Hin*dIII+*Eco*RI and *Hin*dIII+*Spe*I digestion, respectively, ligated with T4 polymerase and re-digested with *Eco*RI. The resulting 1.25-kb DNA fragment was cloned into the *Eco*RI site of pEXGm5B. The ensuing plasmid was digested with *Hin*dIII and ligated with the 1.5-kb *FRT*-*npt*II-*FRT* *Hin*dIII fragment from pFKM2 containing the Km^R^ marker to create pEXGm5Δ*pchA*::FRTKm. This plasmid was transferred to 1710b, Bp327 and Bp338 by conjugation and merodiploids were selected on Km-containing LB plates. Transformation of merodiploid isolates with pBADSce, followed by simultaneous sucrose counter-slection, arabinose induction of I-*Sce*I counter-selection, selection of Km^R^ colonies, and pBADSce curing resulted in the respective Δ*pchA*::FRTKm mutant derivatives. The Km^R^ marker was removed using Flp recombinase . The presence of the mutation in the strains Bp447, Bp448 and Bp449 was confirmed by Southern blot analysis of EcoRI digests of the respective chromosomal DNAs using the 0.5-kb *pchA* 5’ DNA fragment as the probe.

**Bp486 and Bp487.** A 0.85-kb *pchC*  fragment was PCR amplified from 1710b genomic DNA with primers 2152+2153 and cloned into pCR2.1. The 0.75-kb *pchA* 3’ and 0.85-kb *pchC* fragments were released from the resulting pCR2.1 clones by digestion with *Hin*dIII+*Spe*I and *Hin*dIII+*Eco*RI, respectively, ligated with T4 polymerase, and re-digested with EcoRI. The ensuing 1.6-kb DNA fragment was cloned into the *Eco*RI site of pEXGm5B. The 1.5-kb *FRT*-*npt*II-*FRT* *Hin*dIII fragment from pFKM2 containing the Km^R^ marker was ligated into the central HindIII between the 5’ and 3’ *pchBA* flanking fragments to create pEXGm5Δ*pchBA*::FRTKm. This plasmid was transferred to 1710b and Bp338 by conjugation and merodiploids selected on Km-containing LB plates. Sucrose counter-selection and selection for Km^R^ yielded the respective Δ*pchBA*::FRTKm mutant derivatives of 1710b (Bp486) and Bp338 (Bp487). Bp487 candidates were also identified by their Gm^S^ phenotypes. The presence of the correct deletions in the mutant strains was confirmed by PCR with primers 1656+2214, 1597+2214, 1656+2130, and 2212+2213, followed by sequencing of the resulting PCR fragment with primer 2130.

**Bp515 and Bp516.** A 0.85-kb *fptA* 3’ fragment was PCR-amplified from 1710b genomic DNA with primers 2215+2070 and cloned into pCR2.1. The 0.75-kb *pchA* 3’ fragment (obtained during construction of Bp447) and 0.85-kb *fptA* 3’ were released from the resulting pCR2.1 constructs by digestion with *Hin*dIII+*Spe*I and *Hin*dIII+*Eco*RI, respectively, ligated with T4 polymerase and re-digested with *Eco*RI. The ensuing 1.6-kb DNA fragment was cloned into the *Eco*RI site of pEXGm5B. The 1.5-kb *FRT*-*npt*II-*FRT* *Hin*dIII fragment from pFKM2 containing the Km^R^ marker was ligated into the central HindIII between the *pchA* and *ftpA* flanking fragments to create to create pEXGm5ΔPCH::FRTKm. This plasmid was transferred to 1710b and Bp338 by conjugation and merodiploids selected on Km-containing LB plates. Sucrose counter-selection in the presence of Km yielded the respective ΔPCH::FRTKm mutants. Bp516 mutant candidates were also identified by their Gm^S^ phenotypes. The Km^R^ marker was then removed using Flp recombinase following a previously described protocol . The presence of the correct deletion in mutant strains was confirmed by PCR with primers 536+537, 1655+2070, and 2212+2213, followed by sequencing of the resulting PCR fragment with primer 2130.

**Bp549.** Two DNA fragments of 0.8-kb and 0.6-kb corresponding to the left and right flanking regions of the HMU locus as depicted in Fig. 4 were PCR-amplified from 1710b genomic DNA with primers 2273+2274 and 2275+2276, respectively, and cloned into pCR2.1. The DNA fragments corresponding to the left and right HMU flanking regions were released from the resulting pCR2.1 clones by digestion with *Hin*dIII+*Eco*RV and *Hin*dIII+*Eco*RI, respectively, ligated with T4 polymerase and then re-digested with EcoRI. The ensuing 1.4-kb product was cloned into the *Eco*RI site of pEXGm5B. The 1.5-kb *FRT*-*npt*II-*FRT* *Hin*dIII fragment from pFKM2 containing the Km^R^ marker was ligated into the central *Hin*dIII site between the two HMU flanking regions to create pEXGm5ΔHMU::FRTKm. This plasmid was transferred to Bp516 by conjugation and merodiplids selected on LB plates containing Km. Sucrose counter-selection in the presence of Km and X-gluc resulted in a ΔHMU::FRTKm mutant. White colonies were tested for their Km^R^ Gm^S^ phenotypes, followed by removal of the Km^R^ marker using Flp recombinase . The correct mutant genotype was confirmed by PCR with primers 2297+2213 and 2296+2297, followed by sequencing of the resulting PCR fragment with primer 2296.

**Bp568.** Two DNA fragments of 1.1-kb and 0.85-kb corresponding to the left and right flanking regions of the HEM locus as depicted in Fig. 4 were PCR amplified from 1710b genomic DNA with primers 2304+2305 and 2300+2301, respectively, and cloned into pCR2.1. The DNA fragments corresponding to the left and right HEM flanking regions were released from the resulting pCR2.1 constructs by digestion with *Hin*dIII+*Spe*I and *Hin*dIII+*Eco*RI, respectively, ligated with T4 polymerase and re-digested with *Eco*RI. The ensuing 1.95-kb DNA fragment was then cloned into the *Eco*RI site of pEXGm5B. The 1.5-kb *FRT*-*npt*II-*FRT* *Hin*dIII fragment from pFKM4 containing the Km^R^ marker was ligated into the central *Hin*dIII site between the two HEM flanking regions to create pEXGm5ΔHEM::FRTKm. This plasmid was transferred to Bp549 by conjugation and merodiploids selected on LB plates containing Km+Gm. Sucrose counter-selection on Km-containing medium yielded the ΔHEM::FRTKm mutant. White colonies were tested for Km^R^ Gm^S^ phenotypes, followed by removal of the Km^R^ marker using Flp recombinase . The HEM deletion genotype was confirmed by PCR with 1) primers 2213+2332, followed by sequencing with primer 2213, and 2) primers 2331+2332, followed by sequencing with primer 2331.

**References**

1. Lopez CM, Rholl DA, Trunck LA, Schweizer HP (2009) Versatile dual-technology system for markerless allele replacement in *Burkholderia pseudomallei*. Appl Environ Microbiol 75: 6496-6503.

2. Choi K-H, Mima T, Casart Y, Rholl D, Kumar A, et al. (2008) Genetic tools for select agent compliant manipulation of *Burkholderia pseudomallei*. Appl Env Microbiol 74: 1064-1075.

3. Trunck LA, Propst KL, Wuthiekanun V, Tuanyok A, Beckstrom-Sternberg SM, et al. (2009) Molecular basis of rare aminoglycoside susceptibility and pathogenesis of *Burkholderia pseudomallei* clinical isolates from Thailand. PLoS Negl Trop Dis 3: e0000519.

4. Wang RF, Kushner SR (1991) Construction of versatile low-copy-number vectors for cloning, sequencing and gene expression in *Escherichia coli*. Gene 100: 195-199.
